# Clinical course of proteinuria due to cubilin variants: a large multicenter pediatric cohort

**DOI:** 10.1007/s00467-026-07227-4

**Published:** 2026-03-04

**Authors:** Neslihan Cicek, Ceren Alavanda, Ayse Seda Pınarbası, Aylin Inal, Bahriye Atmıs, Secil Kezer, Mehtap Akbalik Kara, Okan Akaci, Serim Pul, Sevcan Hatipoglu, Zeynep Nagehan Yuruk Yildirim, Ayse Agbas, Emre Leventoglu, Ilknur Girisgen, Mehtap Kaya, Nimet Sasmaz Nurdag, Sercin Guven, Ozde Nisa Turkkan, Nurdan Yildiz, Pinar Ata, Ismail Dursun, Ibrahim Gokce

**Affiliations:** 1 Department of Pediatric Nephrology, Marmara University School of Medicine, Fevzi Çakmak Mahallesi Muhsin Yazıcıoğlu Caddesi No:10, Ust Kaynarca, Pendik Istanbul, Türkiye; 2 Department of Medical Genetics, Istanbul Faculty of Medicine, Istanbul University, Istanbul, Türkiye; 3 Department of Pediatric Nephrology, Diyarbakir Childrens’ Hospital, Diyarbakır, Türkiye; 4 Department of Pediatric Nephrology, Faculty of Medicine, Erciyes University, Kayseri, Türkiye; 5 Department of Pediatric Nephrology, Faculty of Medicine, Cukurova University, Adana, Türkiye; 6 Department of Pediatric Nephrology, Bagcılar Training and Research Hospital, Istanbul, Türkiye; 7 Department of Pediatric Nephrology, Faculty of Medicine, Gaziantep University, Gaziantep, Türkiye; 8 Department of Pediatric Nephrology, University of Health Sciences, Bursa Yuksek Ihtisas Training and Reseach Hospital, Bursa, Türkiye; 9 Department of Pediatric Nephrology, Umraniye Training and Research Hospital, Istanbul, Türkiye; 10 Department of Pediatric Nephrology, Faculty of Medicine, Dokuz Eylül University, Izmir, Türkiye; 11 Department of Pediatric Nephrology, Istanbul Faculty of Medicine, Istanbul University, Istanbul, Türkiye; 12 Department of Pediatric Nephrology, Cerrahpaşa Faculty of Medicine, Istanbul University, Istanbul, Türkiye; 13 Department of Pediatric Nephrology, Konya City Hospital, Konya, Türkiye; 14 Department of Pediatric Nephrology, Faculty of Medicine, Pamukkale University, Denizli, Türkiye; 15 Department of Pediatric Nephrology, University of Health Sciences Kartal Dr. Lutfi Kırdar City Hospital, Istanbul, Türkiye; 16 Department of Medical Genetics, Marmara University School of Medicine, Istanbul, Turkey

**Keywords:** Children, Cubilin, *CUBN*, Proteinuria

## Abstract

**Introduction:**

We aimed to evaluate the clinical and genetic characteristics and clinical course of children with persistent proteinuria associated with *CUBN* variants.

**Methods:**

Forty-eight children with *CUBN* variants from 15 pediatric nephrology centers were included. Patients’ characteristics, serum creatinine, albumin, hemoglobin, vitamin B12 levels, urinalysis, spot urine protein/creatinine (uPCR), microalbumin/creatinine (uACR), beta-2 microglobulin/creatinine (uBMCR) ratios, estimated glomerular filtration rates (eGFRs), treatments, kidney biopsies, and genetic findings were evaluated.

**Results:**

All patients had normal serum albumin and creatinine and preserved eGFR. There was no significant change in eGFR between the first and last visits (*p* = 0.15), whereas uPCR was lower at the last visit (*p* = 0.001). Kidney biopsy was performed in 13 (27%); light microscopy was normal in all except one patient with focal segmental glomerulosclerosis (FSGS). Thirty-five patients (72.9%) had ACEi/ARB therapy, which was discontinued in 21 patients without subsequent worsening of proteinuria. Overall, 26 distinct *CUBN* variants were identified, predominantly in the C-terminal region. The most frequent variant was c.10102A > G (p.Met3368Val). Variant types included 15 (57.7%) missense, 7 (27%) nonsense, 3 (11.5%) splicing, and 1 (3.8%) frameshift variants.

**Conclusions:**

In this multicenter, large pediatric cohort, proteinuria associated with *CUBN* variants generally followed a benign course over short to mid-term follow-up even without sustained ACEi/ARB therapy. Embedding targeted *CUBN* testing into the evaluation of children with asymptomatic proteinuria and normal kidney function may reduce unnecessary kidney biopsies and prolonged medication, while improving family counseling. We outline a stepwise care pathway → early genetic screening → conservative monitoring with periodic eGFR and proteinuria assessment → consideration of ACEi/ARB discontinuation and recommend prospective validation and cost-effectiveness studies.

**Graphical abstract:**

**Figure:**
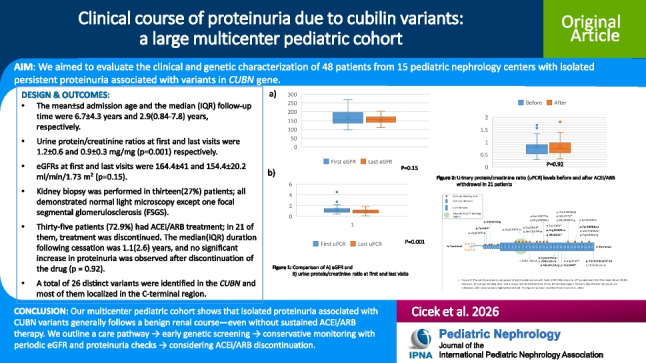
A higher resolution version of the Graphical abstract is available as Supplementary information

**Supplementary Information:**

The online version contains supplementary material available at 10.1007/s00467-026-07227-4.

## Introduction

Variants in *CUBN* gene are associated with Imerslund–Gräsbeck syndrome (IGS), a rare autosomal recessive disorder characterized by selective intestinal malabsorption of vitamin B12, typically presenting with childhood-onset megaloblastic anemia that responds to parenteral vitamin B12 therapy. Mild proteinuria is frequently, but not always present [1, 2]. *CUBN* variants have also been identified as a rare cause of chronic benign proteinuria (PROCHOB) which is characterized by sub-nephrotic-range proteinuria without hypoalbuminemia or kidney dysfunction [3]. While disease-causing variants linked to IGS are more often located in the N-terminal region of the cubilin, more recent studies have identified variants in the C-terminal domain in individuals presenting with PROCHOB [4–7]. Based on limited published cohorts, proteinuria due to *CUBN* variants has generally been associated with a favorable prognosis [4, 5, 8]. In this multicenter study, we evaluated 48 patients with persistent proteinuria and *CUBN* variants along with detailed genotype and phenotype descriptions.

## Methods

This retrospective, multicenter study included patients who underwent genetic testing for proteinuria, were found to harbor a *CUBN* variant, and had complete clinical data available. Clinical data were collected from 15 pediatric nephrology centers in Türkiye and 48 patients were evaluated with a median (IQR) follow-up time of 2.9 (0.84–7.8) (range 0.2–18.8) years in the study.

Written informed consent was obtained from the parents. The study was approved by Marmara University School of Medicine Ethics Committee (09.2025.25–0110). All procedures were conducted in accordance with the ethical standards of the institutional committee and the Declaration of Helsinki.

Patients’ demographic characteristics, serum creatinine, albumin, hemoglobin, vitamin B12 levels, urinalysis, spot urine protein/creatinine ratio (uPCR), urine microalbumin/creatinine ratio (uACR) and urine beta-2 microglobulin/creatinine ratio (uBMCR); 24-h urine protein and microalbumin excretion and estimated glomerular filtration rate (eGFR) at presentation and at the last visit were evaluated. Doppler urinary system ultrasounds and, if performed, kidney biopsies were also evaluated.

Estimated glomerular filtration rate was calculated using the Schwartz formula with a constant k = 0.55 for children and adolescent girls and k = 0.7 for adolescent boys since serum creatinine was measured with the Jaffe method; and eGFR ≥ 90 mL/min/1.73 m^2^ was considered normal [9]. Proteinuria was defined as uPCR > 0.2 mg/mg and nephrotic range proteinuria as > 2 mg/mg. Microalbuminuria was defined as uACR > 30 mg/g and beta-2 microglobulinuria as uBMCR > 0.5 μg/mg for children < 6 years and > 0.35 μg/mg for children ≥ 6 years. For longitudinal analysis, uPCR, eGFR, serum albumin and hemoglobin level were compared between the initial and the last visits.

### Genetic analysis

Written informed consent was obtained from all participants after collection of clinical histories and comprehensive pedigree analyses. Genomic DNA was extracted from peripheral blood samples using the QIAamp DNA Mini Kit (Qiagen, Hilden, Germany). Targeted sequencing of 44 kidney disease-associated genes, including *CUBN* (NM_001081), was performed using the Sophia Nephropathies Solution (NES) kit on an Illumina NextSeq 500 platform (San Diego, CA, USA). Clinical Exome Sequencing (CES) was carried out using next-generation sequencing on the Illumina NextSeq 500 platform with the Sophia Clinical Exome Solution V3 kit, which includes 5500 genes. Data were analyzed using Sophia DDM-V4® software. In population databases such as ExAC, ESP, 1000 Genomes (1000G), and gnomAD, variants with a minor allele frequency (MAF) ≥ 0.01 were excluded. Remaining variants relevant to the patient’s phenotype were evaluated using ClinVar and the Human Gene Mutation Database (HGMD). Variant classification was performed in accordance with ACMG guidelines [10]. Segregation analysis was conducted on an Illumina MiSeq platform (San Diego, CA, USA).

### Statistical analysis

All data were analyzed using the Statistical Packages for the Social Sciences (SPSS Inc., Chicago, IL, USA) 23.0 package. Continuous variables were assessed for normality using the Shapiro–Wilk test and are presented as mean ± standard deviation (SD) for normally distributed data or median (interquartile range, IQR) for non-normally distributed data. Categorical variables are presented as counts and percentages. Between-group comparisons were performed using Student’s t-test or Mann–Whitney U test, as appropriate. Categorical variables were compared using the chi-square test or Fisher’s exact test. For within-patient comparisons between the first and last visits, paired t-test or Wilcoxon signed-rank test was used, depending on distribution. For each first-to-last visit comparison, analyses were restricted to patients with available paired measurements for that variable, and the corresponding number of patients (n) are reported. Two-sided *p* values < 0.05 were considered statistically significant.

## Results

**Fig. 1:**
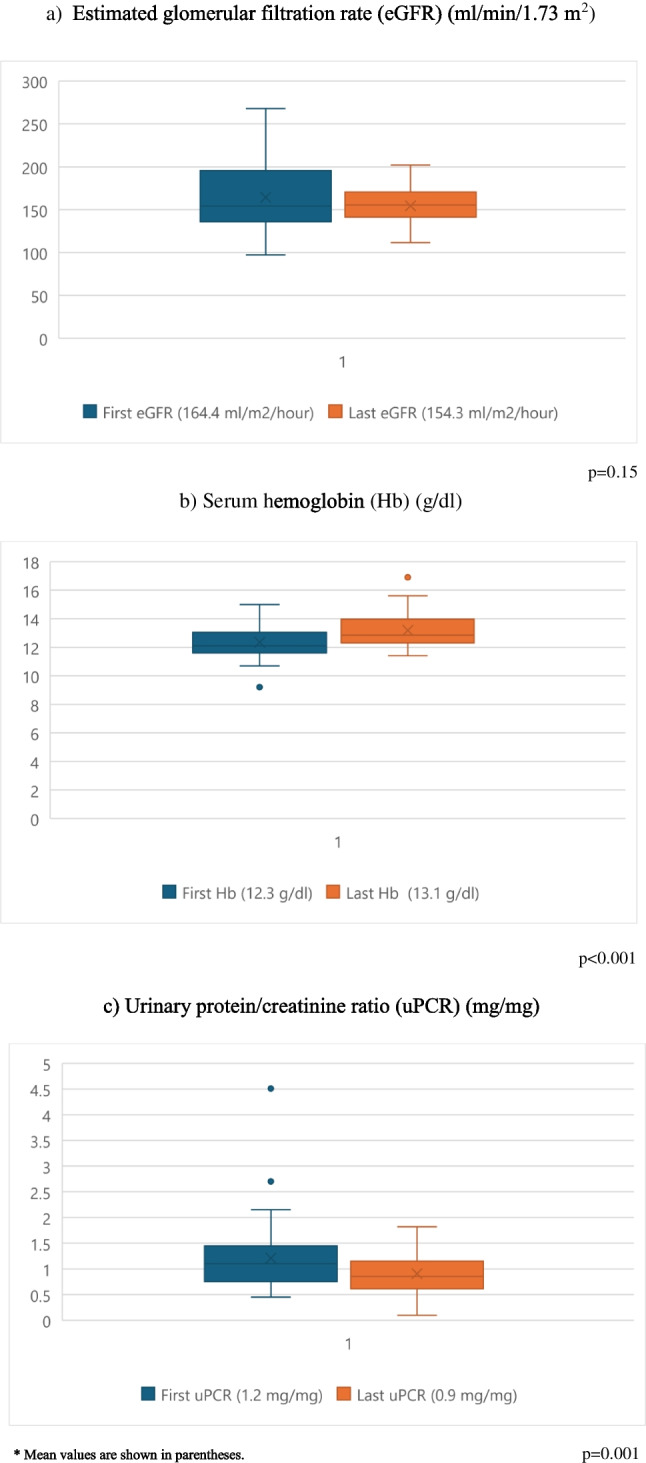
Comparison of eGFR, serum hemoglobin and urine protein/creatinine ratio at first and last visit (*n* = 48)

Forty-eight patients (23 female, 25 male) were evaluated. The mean age at presentation was 6.7 ± 4.3 years and the median follow-up time was 2.9 (0.84–7.8) (range 0.2–18.8) years. Follow-up time exceeded ten years in nine (19%) patients and was between 5–10 years in eleven (23%) patients. Consanguinity was present in 31 (64.6%) patients, and seven patients belonged to three families (Table 1). Forty-five (93.8%) patients were referred for incidentally detected persistent proteinuria, and three (6.2%) patients were screened due to an index case in the family. Serum albumin, serum creatinine, and eGFR were within normal ranges in all patients. Urinalysis showed no hematuria at admission or at the last visit. Complement levels (C3 and C4) were measured in 41 (85.4%) patients and were normal in all. ANA and anti-DNA were assessed in 34 (70.8%) patients; results were negative in 31 and weakly positive (1 +) in 3 patients. At admission the mean uPCR was 1.2 ± 0.6 mg/mg (range 0.4–2.1). The mean 24-h urinary protein excretion was 18.7 ± 8.2 mg/m^2^/hour (range 190–1175 mg/day), uACR was elevated in all patients with a mean of 477.5 ± 184 mg/g (150–860). Urine BMCR was evaluated in 22 (45.8%) patients and was in normal ranges in all with a mean of 0.23 ± 0.2 μg/mg. Urine PCR at the last visit was 0.9 ± 0.3 mg/mg (0.3–1.2). Four patients (8.3%) had nephrotic range proteinuria at presentation, in all of whom proteinuria decreased to subnephrotic levels at the last visit. One of these four patients underwent a kidney biopsy with normal findings and had been treated with corticosteroids and cyclosporine. Two of the four (including this patient) are still on ACEi therapy, while the other two are followed conservatively without medication. At the last visit, all patients had non-nephrotic range proteinuria, and the last uPCR was lower than the first uPCR (*p* = 0.001). There were no significant differences in eGFR and serum albumin levels between first and last visits (*p* = 0.15 and 0.074, respectively). Serum hemoglobin was higher at the last visit (12.3 ± 1.2 vs. 13.1 ± 1.3) (*p* < 0.001) (Fig. 1). Serum vitamin B12 was low in 10 (20.8%) patients and increased to normal ranges after oral vitamin B12 treatment in eight and parenteral treatment in two patients (Table 1). Variants in patients responding to oral therapy were located closer to the C-terminal region, whereas variants in the two patients requiring parenteral B12 were closer to the N-terminal region. Hemoglobin levels were 10.8 and 11.7 g/dl, and MCV values 65 and 79 fl in these two patients.

**Table 1:** Demographic and clinical characteristics of patients with CUBN variants

Characteristics	All patients *n* = 48
Female/male, *n* (%)	23 (47.9%)/25 (52.1%)
Age at admission, mean ± sd (years)	6.7 ± 4.3
Folllow up time, median (IQR) (min–max) (years)	2.9 (0.84–7.8) (0.2–18.8)
Parental consanguinity, *n* (%)	31 (64.6%)
**At first visit**	
Vitamin B12 deficiency (< 180 pg/ml), *n* (%)	10 (20.8%)
uPCR (mg/mg), mean ± sd	1.2 ± 0.6
uACR (mg/g), mean ± sd	477.5 ± 184
uBMCR (μg/mg)	0.23 ± 0.2
Serum albumin (g/dl), mean ± sd	4.5 ± 0.38
eGFR (ml/min/1.73 m^2^), mean ± sd	164.4 ± 41
Hemoglobin (g/dl), mean ± sd	12.3 ± 1.2
Kidney biopsy, n (%)	13 (27%)
Histopathology	1 FSGS, 12 normal
Treatment, n (%)	
ACEi ACEi + ARB	35 (72.9%) 3 (6.3%)
Cessation of ACEi/ARB	21 (60%)*
Immunsupressive treatment, n (%)	
Corticosteroid Cyclosporine Rituximab	2 (4.2%) 2 (4.2%) 1 (2.1%)
**At last visit**	
uPCR (mg/mg), mean ± sd	0.9 ± 0.3
Serum albumin (g/dl), mean ± sd	4.6 ± 0.34
eGFR (ml/min/1.73 m^2^), mean ± sd	154.4 ± 20.2
Hemoglobin (g/dl), mean ± sd	13.2 ± 1.3

*ACEi* angiotensin-converting enzyme inhibitors; *ARB* angiotensin receptor blocker; *eGFR* estimated glomerular filtration rate; *uACR* urine microalbumin/creatinine ratio; *uBMCR* urine beta-2 microglobulin/creatinine ratio; uPCR: urine protein/creatinine ratio

*percentage in patients on ACEi/ARBs treatment

Doppler ultrasonography was normal in all patients. Kidney biopsy was performed in 13 (27%) patients, and light microscopy demonstrated normal pathology or minimal increased mesangial matrix and negative immunofluorescence examination in all except one patient diagnosed with FSGS. Two biopsies were performed three years apart in this patient. The first biopsy demonstrated one globally sclerotic glomerulus out of 50, while the second revealed periglomerular fibrosis in one glomerulus among 27. Immunofluorescence was negative, and electron microscopy showed obliteration of epithelial cells. Based on these histopathological findings, the patient was diagnosed with FSGS. Thirty-five (72.9%) patients had ACEi and three of them received combined ACEi and angiotensin receptor blocker (ARB) therapy, whereas two patients had additional immunosuppressive treatments. (Table 1). One of these patients was the patient with FSGS who was treated with corticosteroids, cyclosporine and rituximab. The second patient who had nephrotic range proteinuria at presentation and normal biopsy findings, received corticosteroids and cyclosporine, and remains on ACEi therapy.

**Fig. 2:**
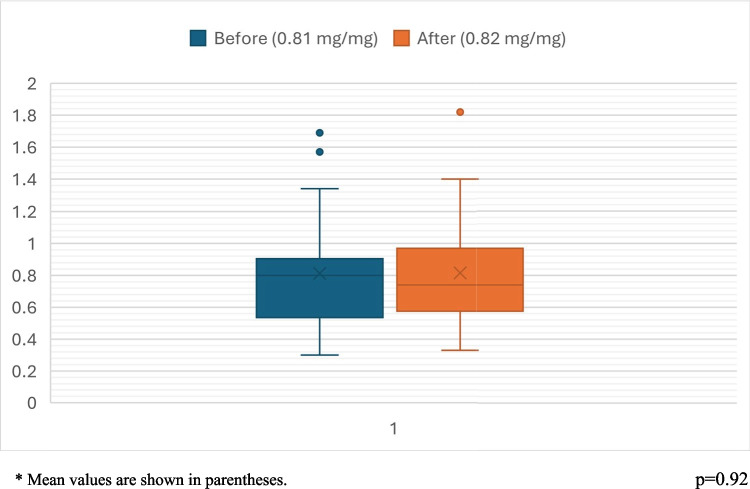
Urinary protein/creatinine ratio (uPCR) levels before and after ACEi/ARB withdrawal in 21 patients

**Fig. 3:**
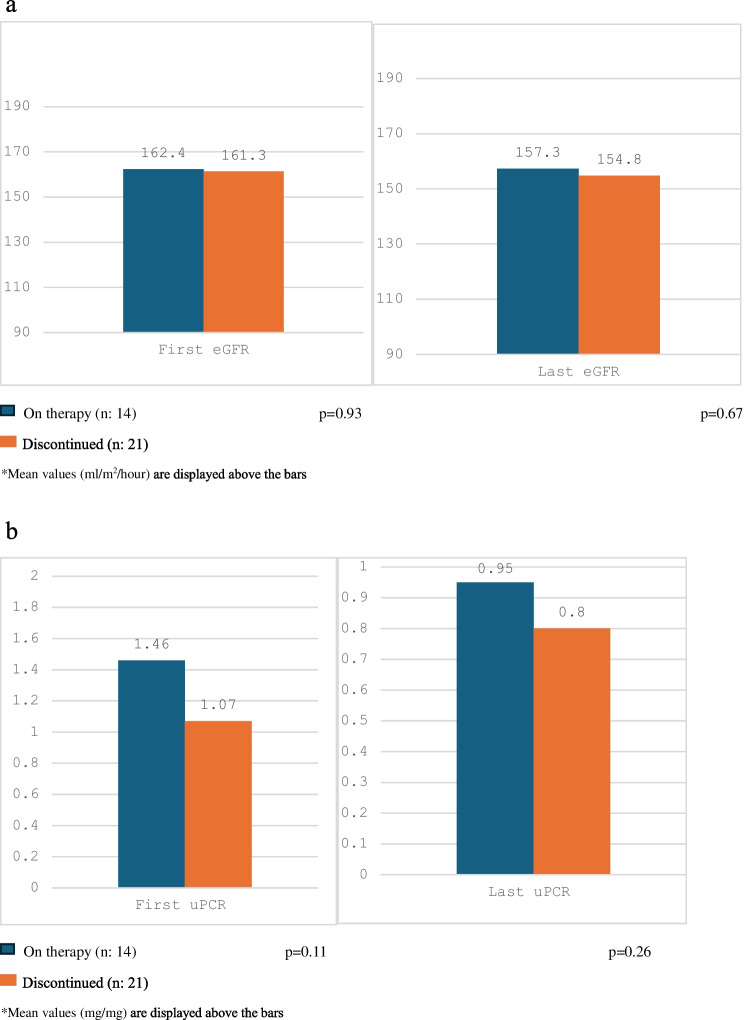
**a**) Estimated glomerular filtration rate (eGFR) in ACEi/ARB therapy group and discontinued group. **b**) Urinary protein/creatinine ratio (uPCR) in ACEi/ARB therapy group and discontinued group

ACEi/ARB therapy was discontinued in 21 patients (60% of those receiving ACEi/ARB). The median (IQR) time since cessation was 1.1 (2.6) years. Proteinuria did not differ significantly before vs. after ACEi/ARB withdrawal (*p* = 0.92) (Fig. 2). We also compared patients who continued ACEi/ARB therapy (n:14) with those who discontinued (n:21) with respect to eGFR and uPCR at the first and last visits; there was no difference in eGFR and uPCR at the first and last visits (*p* = 0.93, 0.67, 0.11, and 0.26, respectively) (Fig. 3).

### Genetic results

**Fig. 4:**
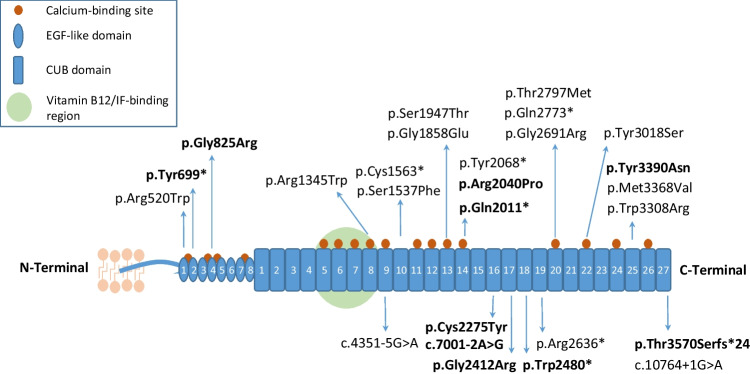
The cubilin protein is composed of eight epidermal growth factor (EGF)-like domains, 27 complement C1r/C1s, Uegf, Bmp1 (CUB) domains, 17 calcium-binding sites, and a single vitamin B12/intrinsic factor (IF)-binding region. Variants identified in this study are indicated, with *novel* variants highlighted in bold. The figure has been modified from Cicek et al. 2023 [8]

In this study, homozygous *CUBN* variants were identified in 38 patients (79.1%), and 10 (20.8%) patients carried compound heterozygous variants ( ). A total of 26 distinct *CUBN* variants were identified (Fig. 4). Fifteen variants (57.7%) were non-truncating and 11 (42.3%) were truncating variants. A detailed examination of the variant types revealed that 15 (57.7%) were missense, 7 (27%) were nonsense, 3 (11.5%) were splicing, and one (3.8%) was frameshift. The most frequently detected variant was c.10102A > G (p.Met3368Val), identified in 18 patients; 16 (88.8%) in the homozygous state and 2 (11.1%) in the compound heterozygous state. In terms of exon distribution, the most frequently affected exon was exon 63, where recurrent variants such as p.Met3368Val and p.Tyr3390Asn were located. Other commonly affected regions included exons 52, 57, and 31. Of the 26 different variants, 4 (15.3%) were located closer to N-terminal region, and 22 (84.6%) to C-terminal region. Segregation analysis was performed in 14 families, and confirmed heterozygous carrier status in both parents in all.

## Discussion

To the best of our knowledge, this study represents the first large pediatric cohort, comprising 48 patients with proteinuria associated with *CUBN* variants, and expands the current understanding of the phenotypic spectrum. Approximately half of the cohort had follow-up exceeding 5 years, including a subset followed for more than 10 years. The most frequently detected variant was *c.10102A* > *G (p.Met3368Val)* and we suggest that this variant could be a Turkish founder variant; the fact that it is rarely reported in large patient series from other countries lends additional support [4, 11]. Kidney histology was unremarkable in 13 patients, except for one case demonstrating FSGS. Most patients received ACEi/ARB, yet proteinuria did not worsen after withdrawal in a substantial proportion, and kidney function remained preserved throughout follow-up, supporting a generally stable clinical course.

Cubilin is a multifunctional endocytic receptor expressed in podocytes, proximal tubular cells, and intestinal epithelium. It plays a key role in albumin reabsorption by the proximal tubule and mediates intestinal absorption of the intrinsic factor–vitamin B12 complex [12]. Evidence from experimental models indicates that a minimal fraction of plasma albumin passes through the glomerular filtration barrier and is subsequently retrieved within the proximal tubular epithelial cells via receptor-mediated endocytosis. This pathway depends on the cooperative function of megalin and cubilin, together with amnionless (the CUBAM complex) at the apical surface of proximal tubular cells. Cubilin, encoded by the *CUBN* gene, is the primary binding receptor for albumin and is therefore considered a key determinant of albumin reclamation [12–14].

In the context of differential diagnosis of proteinuria, it is important to recognize the disorders caused by defective tubular reabsorption of filtered proteins typically present with low-molecular-weight proteinuria [15]. Unlike glomerular proteinuria, tubular proteinuria does not usually require specific therapeutic intervention. Therefore, excluding proteinuria of tubular origin is crucial before initiating immunosuppressive therapy. Recent studies have added *CUBN* defects to the spectrum of genetic disorders causing tubular proteinuria; however, unlike classical tubular disorders, *CUBN*-related disease is distinguished by the predominance of albuminuria. Sakakibara and Nozu recently summarized tubular proteinuria due to hereditary proximal tubular endocytic receptor disorders within a broader differential that includes Dent disease and PROCHOB due to *CUBN* variants. The detailed phenotype of PROCHOB was revealed as no hypoalbuminemia, no kidney dysfunction, and sub-nephrotic-range proteinuria of approximately 0.5 to 1.5 g/gCr, with a lack of response to RAS inhibitors. However, unlike in Dent disease, urinary β2-microglobulin and α1-microglobulin levels remain normal, so these urinary findings resemble glomerular proteinuria, but the proteinuria in PROCHOB is actually tubular proteinuria [3].

Proteinuria is increasingly recognized not only as a marker of glomerular barrier dysfunction but also as a mediator of tubulointerstitial injury. In classical glomerular proteinuric states, filtered proteins and complement components may trigger injurious pathways in proximal tubular epithelial cells, including complement activation at the apical surface [16]. Moreover, filtered albumin is not biologically inert: following endocytosis by proximal tubular cells, albumin can trigger oxidative stress-related signaling, including Rac1-dependent NADPH oxidase activation and reactive oxygen species generation, thereby promoting inflammatory and profibrotic responses [17]. The filtered proteome may also contain bioactive factors released from injured podocytes; for example, podocyte-derived soluble RARRES1 has been shown to accelerate kidney disease progression by inducing direct podocyte injury and proximal tubular damage after tubular uptake [18]. In contrast, in *CUBN*-related albuminuria, the primary defect is impaired cubilin-mediated albumin binding and internalization. This may attenuate intracellular “protein-overload” toxicity pathways that depend on tubular endocytosis, providing a plausible mechanistic explanation for preserved kidney function and minimal tubulointerstitial injury reported in most cohorts, consistent with our findings.

The binding site for albumin is located within the C-terminal domain of cubilin, whereas IGS is more commonly associated with variants affecting the N-terminal region containing the vitamin B12-related binding domain [4, 11]. Consistent with previous reports, most variants in our cohort were located in the C-terminal region. Ten patients had low levels of serum vitamin B12 which normalized after oral treatment in eight, which excludes IGS with no response to oral treatment. Two patients with variants closer to the N-terminal region required parenteral B12 therapy. Hemoglobin levels were somewhat lower than the remainder of the cohort in these two patients; however, they did not have overt anemia, and their MCV values were not elevated, which is inconsistent with macrocytic anemia.

Since the initial report by Ovunç et al. published reports have generally suggested a favorable prognosis in patients with proteinuria associated with *CUBN* variants [4–7, 19, 20]. In our earlier report, six patients followed for a mean of 6.5 years had preserved kidney function and showed no worsening of proteinuria after ACEi/ARB withdrawal [8]. The present series is, to our knowledge, the largest pediatric cohort reported to date and further supports the favorable clinical course of *CUBN*-related proteinuria. Nevertheless, abnormal biopsy findings, including FSGS, have been described in some individuals with *CUBN* variants [8, 21, 22]. In a review by Choi et al. among 74 reported cases, biopsy was performed in 35; one had minimal change disease and six had FSGS. Except for one patient with FSGS and comorbidities (type 2 diabetes and obesity), all patients demonstrated preserved kidney function at last follow-up [5]. Yang et al. reported three pediatric cases presenting with preserved kidney function but biopsy-proven FSGS, each carrying at least one truncating *CUBN* variant. In our cohort, the single patient with an FSGS lesion had limited involvement on both biopsies and stable kidney function with moderate proteinuria over 15 years, raising the possibility of an incidental lesion; however, emerging evidence suggests that FSGS may occur in a subset.

In experimental models, cubilin expression has been documented in human podocytes, and *CUBN* variants may also disrupt albumin handling at the podocyte level, suggesting that albuminuria could reflect combined tubular and glomerular mechanisms in some patients [14, 23]. In addition to the findings at the podocyte level, Bedin et al. reported that their cohort of 39 patients with *CUBN*-associated proteinuria tended to have a more benign disease course, and that some C-terminal missense variants were correlated with higher eGFR values [4]. Consistent with the observations of Bedin et al. in this study, 19 of 34 patients with C-terminal missense variants had elevated eGFR (> 150 mL/min/1.73 m^2^), raising the possibility of hyperfiltration in a subset. Given proposed links between hyperfiltration and glomerulomegaly/FSGS, this may represent one potential pathway; however, prospective studies are required.

Although this study reports a large series of children with *CUBN* variants, its retrospective design and the limited follow-up period to childhood constrain our understanding of the long-term clinical course of the disease and the genotype–phenotype correlations. As a national cohort, generalizability to other populations may be limited due to differences in variant spectrum, ascertainment, and clinical practice. International replication is warranted.

In conclusion, this large pediatric cohort with *CUBN*-associated proteinuria demonstrated preserved kidney function and stable or improving proteinuria over time. Although proteinuria is generally considered as a risk marker for progressive kidney disease, *CUBN* variants may lead to a benign phenotype characterized by persistent proteinuria with preserved kidney function; however, FSGS lesions may be observed in some cases. Based on our findings, we propose a stepwise care pathway: (1) early genetic screening in selected children with asymptomatic proteinuria and normal kidney function, (2) conservative monitoring with periodic assessment of eGFR and proteinuria, and (3) reconsideration of ACEi/ARB discontinuation when clinically appropriate. Prospective studies are needed to validate this approach, define optimal monitoring intervals and treatment thresholds, and evaluate cost-effectiveness and patient-centered outcomes.

## Supplementary Information

Below is the link to the electronic supplementary material.

Graphical abstract (PPTX 182 KB)

## Data Availability

Data is available upon reasonable request.
