# Supplementary material for: Clinical course of proteinuria due to cubilin variants: a large multicenter pediatric cohort
Source: Pediatr Nephrol. 2026 Mar 4;41(7):2089–97. doi: 10.1007/s00467-026-07227-4 (PMC13197286; doi:10.1007/s00467-026-07227-4)
Supplement: Supplementary file 1 — Graphical abstract (PPTX 182 KB) [file 467_2026_7227_MOESM1_ESM.pptx]

## Slide 1
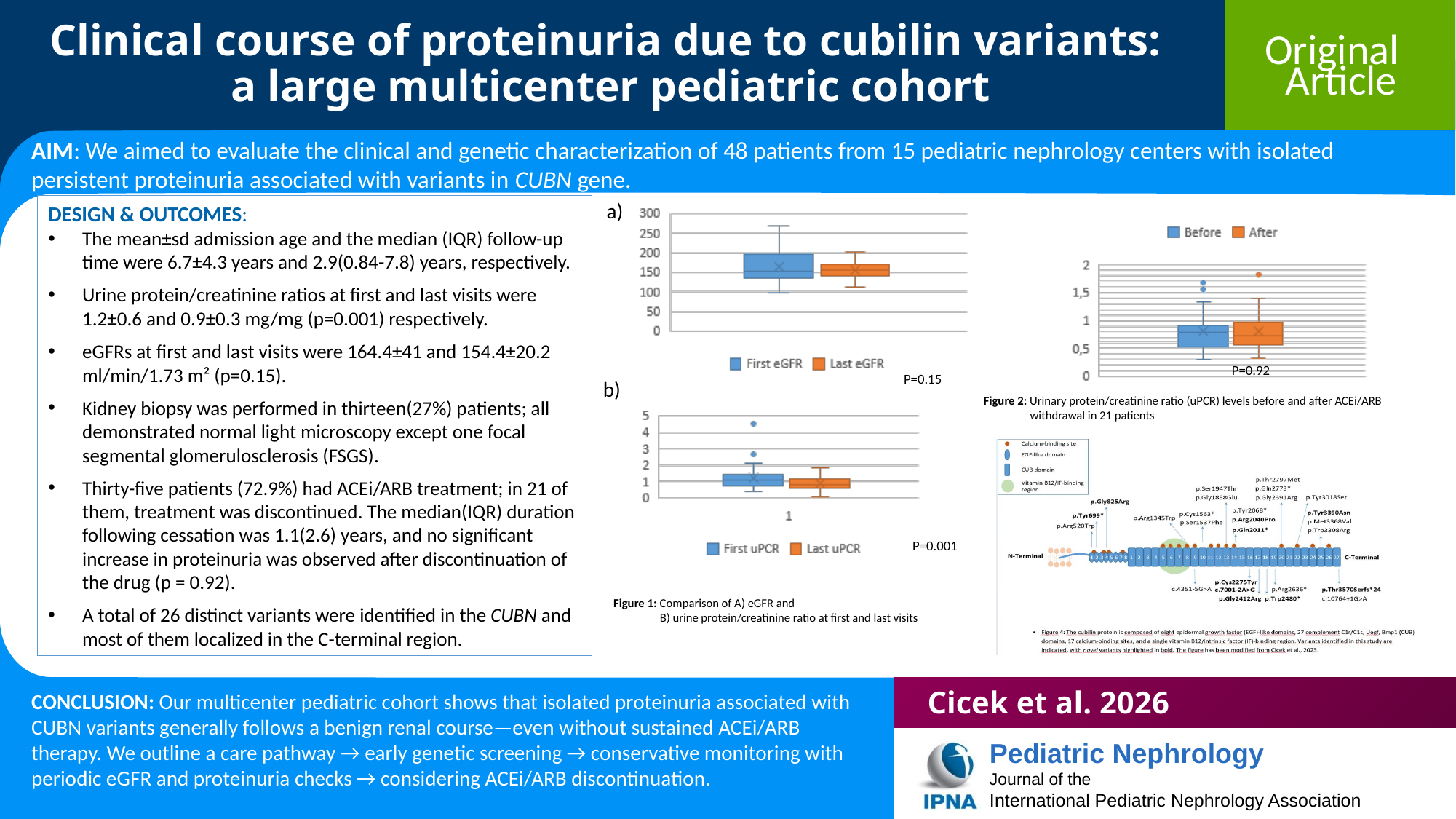

Clinical course of proteinuria due to cubilin variants:
a large multicenter pediatric cohort
AIM: We aimed to evaluate the clinical and genetic characterization of 48 patients from 15 pediatric nephrology centers with isolated persistent proteinuria associated with variants in CUBN gene.
a)
DESIGN & OUTCOMES:
The mean±sd admission age and the median (IQR) follow-up time were 6.7±4.3 years and 2.9(0.84-7.8) years, respectively.
Urine protein/creatinine ratios at first and last visits were 1.2±0.6 and 0.9±0.3 mg/mg (p=0.001) respectively.
eGFRs at first and last visits were 164.4±41 and 154.4±20.2 ml/min/1.73 m² (p=0.15).
Kidney biopsy was performed in thirteen(27%) patients; all demonstrated normal light microscopy except one focal segmental glomerulosclerosis (FSGS).
Thirty-five patients (72.9%) had ACEi/ARB treatment; in 21 of them, treatment was discontinued. The median(IQR) duration following cessation was 1.1(2.6) years, and no significant increase in proteinuria was observed after discontinuation of the drug (p = 0.92).
A total of 26 distinct variants were identified in the CUBN and most of them localized in the C-terminal region.
P=0.92
P=0.15
b)
Figure 2: Urinary protein/creatinine ratio (uPCR) levels before and after ACEi/ARB
 withdrawal in 21 patients
P=0.001
Figure 1: Comparison of A) eGFR and
 B) urine protein/creatinine ratio at first and last visits
Cicek et al. 2026
CONCLUSION: Our multicenter pediatric cohort shows that isolated proteinuria associated with CUBN variants generally follows a benign renal course—even without sustained ACEi/ARB therapy. We outline a care pathway → early genetic screening → conservative monitoring with periodic eGFR and proteinuria checks → considering ACEi/ARB discontinuation.
